# Comparative Transcriptomic Analysis Reveals Key Pathways and Genes Involved in Late-Acting Self-Incompatibility in *Akebia trifoliata*

**DOI:** 10.3390/cimb48030245

**Published:** 2026-02-26

**Authors:** Huai Yang, Jie Li, Rui Han, Xiaoxiao Yi, Chen Chen, Peigao Luo

**Affiliations:** Key Laboratory of Plant Genetics and Breeding at Sichuan Agricultural University of Sichuan Province, College of Agricultural, Sichuan Agricultural University, Chengdu 611130, China

**Keywords:** pollen tube growth, metabolic pathways, reproductive barrier, transcription factors

## Abstract

Self-incompatibility (SI) is a key reproductive mechanism in angiosperms that prevents self-fertilization and promotes genetic diversity while limiting breeding efficiency. *Akebia trifoliata* is a recently domesticated economic crop native to East Asia with medicinal, edible, and oil-producing value. However, its late-acting self-incompatibility (LSI) severely limits genetic improvement and commercial development. To investigate the molecular basis of LSI, we conducted comparative transcriptomic analyses of pistils at 48, 96, 144, 192, and 240 h after self- and cross-pollination, identifying 1552, 2954, 1302, 814, and 1978 differentially expressed genes (DEGs), respectively. DEGs were consistently enriched in mitogen-activated protein kinase (MAPK) signaling, plant hormone signal transduction, and ubiquitin-mediated proteolysis pathways, with clear transcriptional differences before and after 96 h. Compared with cross-pollinated pistils, self-pollinated pistils showed restricted pollen tube spread, and genes related to pollen recognition and tube development showed differential expression at 48 and 96 h, indicating that LSI probably occurs within the pollen tube. Collectively, these results indicate that pistils of *A. trifoliata* exhibit distinct early responses to self- and cross-pollination, and that DEG-enriched pathways are similar to those involved in *S-RNase*-mediated SI. These results provide new insights into the molecular basis of LSI and suggest potential targets for overcoming the SI barrier.

## 1. Introduction

The bisexual floral structure of angiosperms greatly enhances pollination efficiency; however, it simultaneously increases the risk of inbreeding depression resulting from self-pollination [1]. To mitigate this risk, plants have evolved self-incompatibility (SI), a molecular recognition system that inhibits self-pollination while promoting cross-pollination, thereby increasing population diversity and enhancing environmental adaptability [2]. SI systems are generally categorized into two types according to the mode of interaction between pollen and pistil. In gametophytic self-incompatibility (GSI), pollen tube growth is allowed only when the haploid genotype of the pollen differs from that of the pistil. This mechanism is widely distributed across the *Papaveraceae* and several core eudicot families [3]. In sporophytic self-incompatibility (SSI), pollen germination and tube growth can occur when the pollen coat genotype (inherited from the parents) differs from that of the pistil [4,5]. This mechanism is predominantly observed in the *Brassicaceae* family. In contrast, a distinct yet widely distributed form of late-acting self-incompatibility (LSI) has been reported across diverse angiosperm lineages, including monocots, basal eudicots, and core eudicots [6]. In LSI systems, self-pollen tubes usually reach the embryo sac or even penetrate the ovule but fail to achieve fertilization or zygote formation. Nevertheless, the molecular regulatory systems and mechanistic basis underlying LSI remain largely unexplored.

*Akebia trifoliata* (commonly called Augmelon) is a perennial vine belonging to the *Akebia* genus in the *Lardizabalaceae* family, native to East Asia [7,8]. The immature fruit has long been used in traditional Chinese medicine for its anti-inflammatory and diuretic properties, and its use has been documented for over a thousand years [9]. The mature fruit pulp is sweet, soft, and rich in free amino acids, vitamin C, reducing sugars, and other nutrients [10], making it a promising new fruit developed and improved in China, South Korea, and Japan [11,12]. In addition, the large number of seeds and high oil content in the mature fruit give this species great potential as an industrial source of edible oil or biofuel [13]. In recent years, as the nutritional and economic value of *A. trifoliata* has become increasingly known, a large-scale cultivation boom has emerged in southern China. However, its LSI severely limits genetic improvement, orchard management, and variety propagation [14].

The molecular mechanisms of GSI and SSI have been extensively characterized, providing an important reference for investigating LSI. LSI exhibits phenotypic similarities to both systems, including the ability of self-pollen to germinate and produce pollen tubes, as observed in GSI [6], while diallel crossing experiments in *Theobroma cacao*, a species exhibiting LSI, indicate SSI-like recognition behavior [15]. Three major molecular regulatory systems have been extensively characterized. The first is the *S-RNase*/*SLFs* locus, in which the female determinant *S-RNase* (*S*-ribonuclease) and the male determinant *SLF* (*S*-locus *F-box*) are tightly linked to control GSI [16,17]. This system is widely found in *Solanaceae* [18], *Plantaginaceae* [19], *Rosaceae* [20], *Rutaceae* [21], and *Cactaceae* [22]. In this system, SLF proteins interact with SSK1 and Cullin1 to form the SCF^SLF^ complex, which mediates the ubiquitination and degradation of non-self S-RNase proteins, leading to cross-compatibility. In contrast, self-pollination results in SI because the complex fails to recognize and degrade self S-RNase proteins [23]. In incompatible pollen tubes, S-RNase suppresses the production of reactive oxygen species (ROS) at the pollen tube tip, decreases calcium ion (Ca^2+^) levels, causes actin depolymerization, and induces programmed cell death (PCD), thereby preventing the pollen tube from reaching the ovule [24]. The second system involves the female determinant *PrsS* and the male determinant *PrpS*, which together form the *S*-locus governing GSI in the *Papaveraceae* family [25,26]. PrsS and PrpS proteins, acting as the ligand and receptor, respectively, trigger a Ca^2+^-dependent signaling network that inhibits pollen tube growth and activates the mitogen-activated protein kinase (MAPK) pathway, leading to PCD and consequently SI [27,28]. The third system, regulated by the female SRK (*S*-locus receptor kinase) and male SCR/SP11 (*S*-locus cysteine-rich protein) determinants in *Brassicaceae*, controls SSI [29,30]. Upon self-pollination, SRK activation triggers a signaling cascade that increases ROS production, leading to inhibition of pollen germination and pollen tube growth [31]. Although these SI systems differ markedly in their molecular components, they share downstream signaling networks involving MAPK cascades and ubiquitin-mediated protein degradation.

Hormonal signaling plays a crucial role in regulating pollen tube growth, a central process in SI. Studies in *Petunia* have revealed significant differences in the levels of ethylene, free indole-3-acetic acid (IAA), abscisic acid (ABA), and cytokinins between compatible and incompatible pollinations [32]. Moreover, in transgenic SI *Arabidopsis* lines, the transcription factor (TF) *ARF3*, expressed in the vascular tissues, enhances the SI response by down-regulating auxin signaling in the stigma epidermis [33]. In *Torrenia fournieri*, ABA can inhibit pollen tube growth in vitro, and its levels significantly decrease after pollination, indicating that ABA negatively regulates pollen tube growth [34]. In *Pyrus* species, exogenous brassinosteroid treatment alleviates the inhibition of pollen tube growth caused by incompatible S-RNases [35]. Additionally, jasmonic acid (JA) and its derivative, methyl jasmonate (MeJA), have been shown to inhibit pollen tube germination and elongation in species such as *Camellia oleifera*, and JA up-regulates key genes involved in JA signaling, further supporting its role in regulating self-pollination inhibition [36]. Collectively, these findings demonstrate that plant hormone signaling pathways are extensively involved in modulating the SI response.

In this study, we performed a comparative transcriptomic analysis using available transcriptome datasets of pistil development in *A. trifoliata* following self- and cross-pollination. In addition, we compared pollen tube morphology and evaluated differentially expression of genes (DEGs) involved in pollen recognition and pollen tube growth between self- and cross-pollinated pistils. Our objective was to explore dynamic changes in biological pathways associated with the LSI response during pistil development under self- and cross-pollination conditions, and to identify key metabolic pathways and genes involved in the LSI process in *A. trifoliata*.

## 2. Materials and Methods

### 2.1. Data Acquisition and Reference Genome Mapping

A total of 30 transcriptomic datasets derived from pistils of *A. trifoliata* were retrieved from the National Genomics Data Center (NGDC) under project accession PRJCA019245 (https://ngdc.cncb.ac.cn/gsa/search?searchTerm=PRJCA019245 (accessed on 28 September 2025)) (Table S1). These datasets corresponded to pistil samples collected at 48, 96, 144, 192, and 240 h after self- and cross-pollination, with three biological replicates for each treatment and time point (run numbers CRR857369–CRR857398). The selected time points span the major stages of the LSI response in *A. trifoliata*, given that pistils from self-pollinated flowers begin to abscise approximately 10 days after pollination. Raw sequencing reads were subjected to quality control to generate clean reads by removing adapter sequences, poly-N regions, and low-quality reads (Phred quality score < 20). The reference genome of *A. trifoliata* (‘Shusen1′) was obtained from NGDC under project accession PRJCA003847 (genome accession number GWHBISH00000000). Clean reads were aligned to the reference genome using HISAT2 (version 2.2.1) [37]. Transcripts were assembled, and known and novel transcripts were identified using StringTie (version 2.1.6) [38]. Gene expression levels were calculated as fragments per kilobase of transcript per million mapped reads (FPKM) for expression pattern visualization and correlation analyses [39].

### 2.2. Assessment of Transcriptome Reliability and Identification of DEGs

To assess the reliability and overall quality of the transcriptomic datasets, we performed principal component analysis (PCA) and pairwise Pearson’s correlation analysis across all 30 samples using FPKM values to evaluate global expression patterns and biological reproducibility among replicates. PCA was used to visualize sample clustering and variation across different pollination treatments and developmental stages, while Pearson’s correlation coefficients quantified the consistency of gene expression profiles among biological replicates. DEGs between self- and cross-pollinated pistils at each developmental stage were identified using the DESeq2 package (version 1.44.0) [40] applied to raw count data. Genes with an adjusted *p*-value < 0.01 and a fold change ≥ 2 were considered significantly differentially expressed. To further explore the biological functions and pathways associated with DEGs, Gene Ontology (GO) and Kyoto Encyclopedia of Genes and Genomes (KEGG) enrichment analyses were conducted using the KOBAS database and clusterProfiler 4.0 software [41,42].

### 2.3. Observation of Pollen Tube Growth and Identification of Genes Regulating Pollen Tube Development

The *A. trifoliata* line L78 used for pollen tube observation was obtained from the germplasm nursery of the Chongzhou Research Station, Sichuan Agricultural University, located in Chongzhou, Sichuan Province, China (103.66° E, 30.56° N). In March 2025, freshly opened flowers were emasculated and bagged to prevent unwanted pollination. When the stigmas became receptive (visibly moist), self- and cross-pollination were performed manually. Pistils were collected on the 6th and 8th days after pollination and fixed in FAA solution (formalin–acetic acid–alcohol) for 12 h. The fixed pistils were then softened in 2 mol/L NaOH solution at 60 °C until they became transparent. Subsequently, the pistils were stained with 0.1% aniline blue solution for 2 h, and pollen tube growth was observed and photographed using a Zeiss Axio Imager M2 upright fluorescence microscope (Jena, Germany). Genes related to pollen tube growth regulation were identified based on the GO enrichment analysis of the DEGs. GO terms specifically associated with pollen development and recognition were retrieved and screened, including pollen tube growth (GO:0009860), pollen tube development (GO:0048868), regulation of pollen tube growth (GO:0080092), recognition of pollen (GO:0048544), and pollen tube guidance (GO:0010183).

### 2.4. Co-Expression Network Analysis

To investigate the potential regulatory relationships between TFs and differentially expressed functional genes involved in SI-related pathways, we first identified differentially expressed TFs from the DEGs detected at 48 h and 96 h after self- and cross-pollination. TF information was obtained from the previously reported genome-wide identification of TFs in *A. trifoliata* [43]. Pearson correlation coefficients (*r*) and the corresponding *p*-values between TFs and functional genes were calculated in R (version 4.5.1) using the cor.test function from the rstatix package (version 0.7.3). To account for multiple testing, *p*-values were adjusted using the Benjamini–Hochberg false discovery rate (FDR) method. Gene pairs with |*r*| ≥ 0.8 and FDR-adjusted *p* < 0.05 were considered significantly correlated. Subsequently, the potential TF-binding motifs within the promoter regions (1000 base pairs upstream of the coding sequence) of functional genes were predicted using the Plant TF Binding Motif Shift and Fimo: Binding Motif Scan plugins in TBtools (version 2.363). Finally, the co-expression network was visualized using Cytoscape (version 3.10.4).

## 3. Results

### 3.1. Assembly and Reliability Assessment of Pistils Transcriptome Data

**Figure 1:**
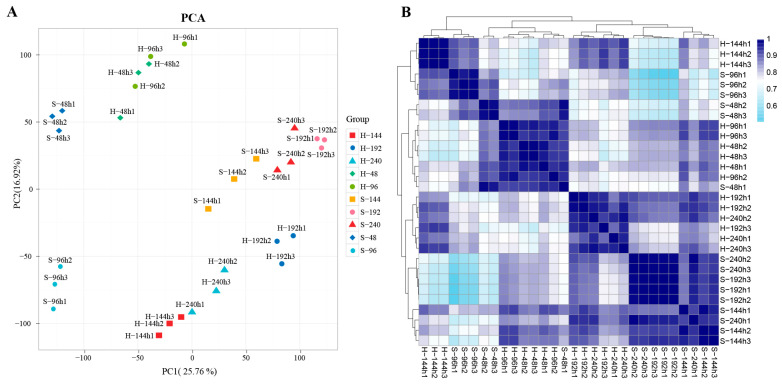
Reliability and reproducibility assessment of transcriptome data (H- represents cross-pollination pistils, and S- represents self-pollinated pistils). (**A**) PCA of 30 transcriptome datasets; (**B**) correlation analysis between transcriptome samples.

A total of 30 transcriptomic datasets from the pistils of *A. trifoliata* at 48 h, 96 h, 144 h, 192 h, and 240 h after self- and cross-pollination (each with three biological replicates) were used for comparative transcriptomic analysis. After sequencing quality control, a total of 1,283,874,024 clean reads were obtained (Table S1). The Q30 base percentage of each sample was no less than 95.18%, and the GC content of clean reads ranged from 43.43% to 43.98% (Table S1). Clean reads from each sample were aligned to the *A. trifoliata* reference genome, with mapping rates ranging from 84.09% to 87.06%, and 4256 putative novel genes were identified. PCA revealed that PC1 and PC2 could clearly separate all samples except those collected at 48 h and 96 h after cross-pollination (Figure 1A), suggesting relatively similar transcriptional profiles between these two early post-pollination stages. In addition, pairwise correlation analysis among biological replicates confirmed the high reproducibility of the data (Figure 1B), demonstrating the overall reliability of the sequencing and analysis results.

### 3.2. Identification of DEGs and Functional Enrichment Analysis

**Figure 2:**
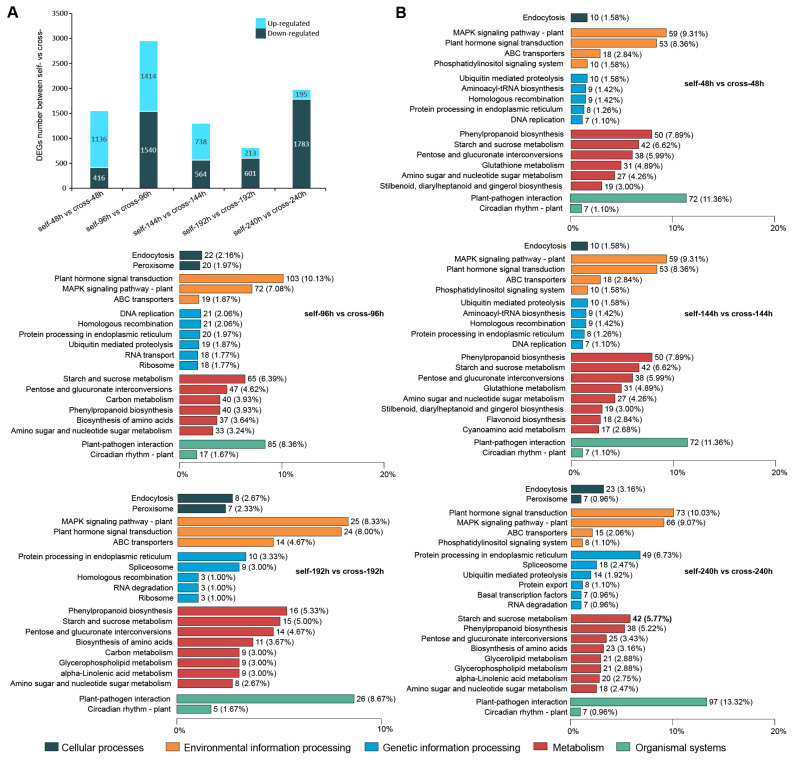
KEGG pathways enriched for DEGs during pistil development in *A. trifoliata* following self- and cross-pollination. (**A**) Number of DEGs identified after self- and cross-pollination. (**B**) Major KEGG pathways enriched for DEGs following self- and cross-pollination.

Differential expression analysis revealed that, compared to cross-pollination, 1552 (1136 up-regulated, 416 down-regulated), 2954 (1414 up-regulated, 1540 down-regulated), 1302 (738 up-regulated, 564 down-regulated), 814 (213 up-regulated, 601 down-regulated), and 1978 (195 up-regulated, 1783 down-regulated) DEGs were identified in pistils 48 h, 96 h, 144 h, 192 h, and 240 h after self-pollination, respectively (Figure 2A).

To further explore the role of DEGs in the pistils of *A. trifoliata* after self- and cross-pollination, KEGG pathway enrichment analysis was performed. A total of 634 (40.85%), 1017 (34.43%), 419 (32.18%), 300 (36.86%), and 728 (36.80%) DEGs were enriched in 113, 126, 98, 110, and 118 pathways at 48 h, 96 h, 144 h, 192 h, and 240 h after self- and cross-pollination, respectively (Table S2). Despite variation in the number of DEGs across comparison groups, the enriched pathways were highly similar (Figure 2B). In the cellular processes category, DEGs were primarily enriched in endocytosis across all time points. In the organismal systems category, DEGs were most enriched in plant–pathogen interaction and circadian rhythm. For environmental information processing, DEGs were predominantly involved in the MAPK signaling pathway, plant hormone signal transduction, and ABC transporters. Regarding genetic information processing, DEGs enriched in specific pathways at different time points were as follows: 48 h: phosphatidylinositol signaling system, ubiquitin-mediated proteolysis, and aminoacyl-tRNA biosynthesis; 96 h: DNA replication, homologous recombination, and protein processing in the endoplasmic reticulum; 144 h: protein processing in the endoplasmic reticulum, DNA replication, and homologous recombination; 192 h: protein processing in the endoplasmic reticulum, spliceosome, and homologous recombination; 240 h: protein processing in the endoplasmic reticulum, spliceosome, and ubiquitin-mediated proteolysis. In the metabolism category, DEGs were primarily enriched in phenylpropanoid biosynthesis and starch and sucrose metabolism across four time points, except at 96 h, where they were mainly enriched in starch and sucrose metabolism and pentose and glucuronate interconversions (Figure 2B).

### 3.3. Changes in the MAPK Signaling Pathway

**Figure 3:**
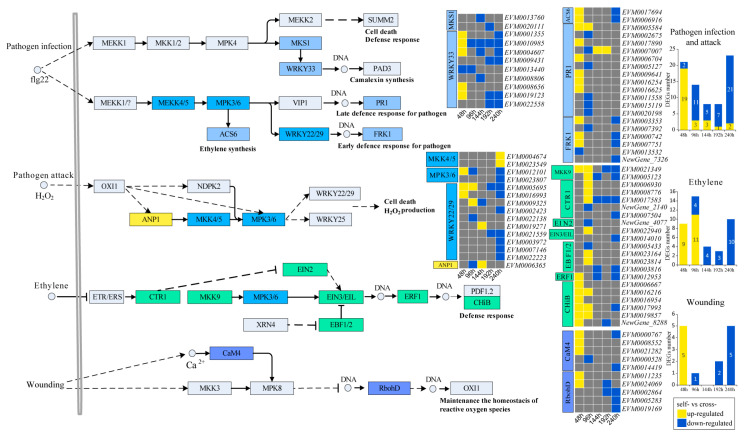
Gene expression changes of four MAPK signaling pathways (The colored modules represent modules where DEGs were detected).

To investigate the changes in the MAPK signaling pathway during the pistil SI response, we compared the expression of four MAPK signaling pathway gene sets potentially involved in SI after self- and cross-pollination. We found that the four MAPK signaling pathway gene sets were up-regulated early after self-pollination (48 and 96 h post-pollination) (Figure 3). In the immune defense pathway induced by pathogen infection and attack, a total of nine modules containing 46 DEGs were detected. Relative to cross-pollination, pistils following self-pollination exhibited 19 up-regulated and two down-regulated DEGs at 48 h. However, at later time points, the majority of DEGs were down-regulated, with 11, 5, 7, and 21 DEGs down-regulated at 96 h, 144 h, 192 h, and 240 h, respectively (Figure 3). The ethylene-induced defense response pathway contained eight modules with 23 DEGs, most of which were up-regulated at 48 h and 96 h after self-pollination. In self-pollinated pistils, all nine DEGs were up-regulated at 48 h, whereas at 96 h, 11 DEGs were up-regulated and 4 were down-regulated. Subsequently, 4, 3, and 10 DEGs were down-regulated at 144 h, 192 h, and 240 h, respectively. In the wounding-related ROS homeostasis pathway, two modules containing 10 DEGs were identified. Five DEGs were up-regulated at 48 h after self-pollination, whereas at subsequent stages, all DEGs were down-regulated in self-pollinated pistils.

### 3.4. Changes in the Plant Hormone Signal Transduction Pathway

**Figure 4:**
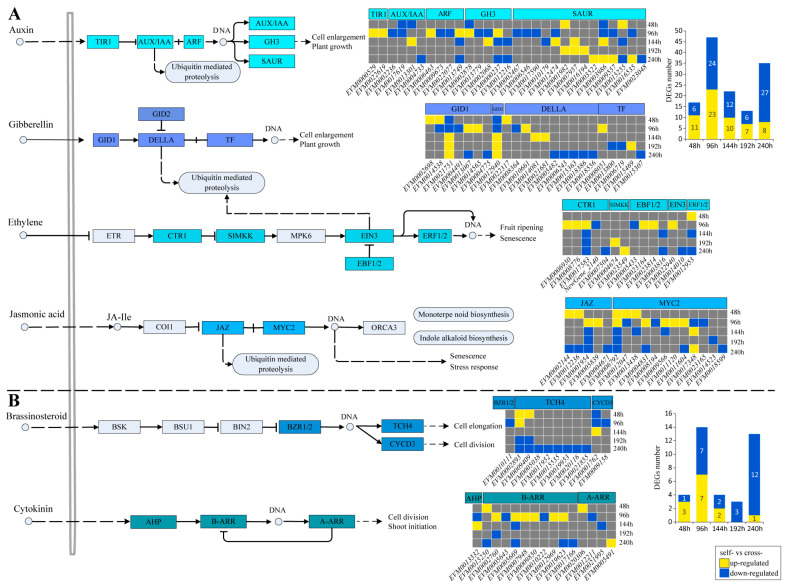
Gene expression differences in hormone signal transduction pathways. The colored modules represent modules where DEGs were detected. (**A**) Four hormone signaling pathways involved in protein ubiquitination and degradation; (**B**) Hormone signaling pathways mediated by brassinosteroid and cytokinin.

We further investigated the expression of plant hormone signal transduction genes potentially involved in SI. The signaling pathways mediated by auxin, gibberellin, ethylene, and JA all regulate protein ubiquitination and degradation. A total of 83 DEGs were detected across these four pathways, most of which were up-regulated at early stages (48 and 96 h) in self-pollinated pistils, followed by a predominance of downregulation at later stages (144, 192 and 240 h) (Figure 4A). Compared to cross-pollinated pistils, self-pollinated pistils exhibited the following differential expression patterns: at 48 h, 11 DEGs were up-regulated and 6 DEGs were down-regulated; at 96 h, 23 DEGs were up-regulated and 24 DEGs were down-regulated; at 144 h, 10 DEGs were up-regulated and 12 were down-regulated; at 192 h, 7 DEGs were up-regulated and 6 DEGs were down-regulated; and at 240 h, 8 DEGs were up-regulated and 27 DEGs were down-regulated (Figure 4A). In signaling pathways regulating cell division, mediated by brassinosteroid and cytokinin, 26 DEGs were detected across six modules (Figure 4B). These DEGs were primarily up-regulated in self-pollinated pistils before 96 h, with 10 genes showing upregulation (Figure 4B). After 144 h, the pathway genes were mostly down-regulated, with 14 genes showing downregulation (Figure 4B).

### 3.5. Expression Analysis of Genes Involved in Ubiquitin-Mediated Proteolysis

Protein ubiquitination plays a crucial role in both SSI and GSI. We focused on the expression of ubiquitination system components related to SI during pistil development after self- and cross-pollination. A total of three differentially expressed components were detected, including one *Cullin* and 14 *F-box* genes from the SCF^SLF^ complex, as well as a *CHIP* gene from the E3 (ubiquitin ligase) ubiquitination system (Table 1). Among these 15 DEGs, 7 DEGs were up-regulated in pistils 48 h after self-pollination, 4 DEGs were up-regulated at 96 h, and these genes showed little to no change at 144 h and 192 h after pollination, while 9 DEGs were down-regulated at 240 h in self-pollinated pistils (Table 1).

### 3.6. The Microscopic Observation of Pollen Tubes and the Expression Analysis of Genes Related to Pollen Tube Growth and Development

**Figure 5:**
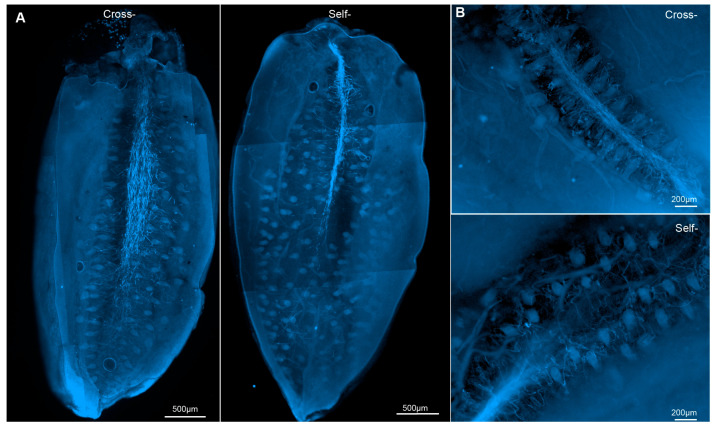
Pollen tube growth and development following self- and cross-pollination. (**A**) Pollen tube development observed 6 days after self- and cross-pollination. (**B**) Pollen tube degradation observed 8 days after self- and cross-pollination.

Microscopic observation of pollen tubes revealed that, in the L78 line, both self- and cross-pollinated pollen tubes reached the vicinity of the embryo sac 6 days after pollination (Figure 5A), consistent with the characteristics of LSI. Morphologically, self-pollinated pollen tubes were more clustered and exhibited a narrower spread compared to cross-pollinated pollen tubes (Figure 5A). However, by 8 days after pollination, the edges of both self- and cross-pollinated pollen tubes became blurred (Figure 5B), indicating the beginning of pollen tube degradation.

Analysis of DEGs regulating pollen recognition and pollen tube growth in pistils following self- and cross-pollination showed that most transcriptional differences occurred at 48 h and 96 h after pollination (Table 2). A total of 24 DEGs associated with pollen recognition were identified. At 48 h after pollination, 11 DEGs were up-regulated and 1 was down-regulated in self-pollinated pistils. At 96 h, five DEGs were up-regulated and eight were down-regulated in self-pollinated pistils. Among the DEGs involved in the regulation of pollen tube growth and development, two DEGs were up-regulated and one DEG was down-regulated at 48 h after self-pollination (Table 2). At 96 h after pollination, two additional DEGs associated with pollen tube growth and development were identified, both of which were down-regulated in self-pollinated pistils (Table 2). Additionally, one DEG related to the regulation of pollen tube growth was found to be up-regulated only at 96 h after self-pollination, while one DEG involved in pollen tube guidance was up-regulated at 96 h and earlier, and then down-regulated at 144 and 240 h (Table 2).

### 3.7. Exploration of TFs Potentially Regulating Functional Genes Involved in SI

**Figure 6:**
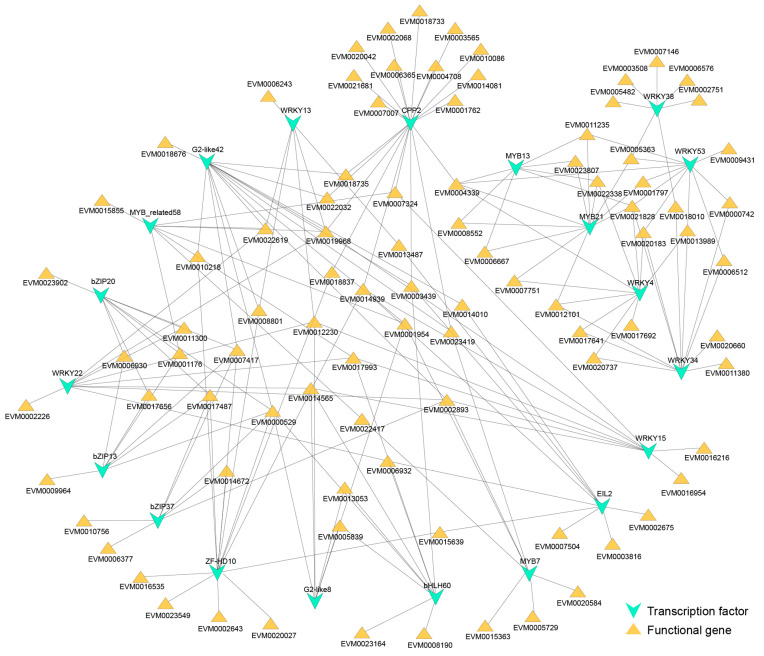
Top 20 TFs and functional genes co-expression network analysis.

To explore the TFs potentially regulating the SI response in *A. trifoliata*, we analyzed 284 differentially expressed TFs identified early after pollination from within the complete set of TFs in the genome, and integrated this analysis with that of 236 functional genes enriched in pathways potentially associated with SI responses, including the MAPK signaling pathway, plant hormone signal transduction, and ubiquitin-mediated proteolysis. By calculating the Pearson correlation coefficients between the expression levels of these TFs and functional genes (|*r*| ≥ 0.8, *p* < 0.05) and integrating the results with promoter-binding site predictions, we identified 100 TFs that may regulate 121 functional genes (Table S3). These TFs exhibited highly correlated expression patterns with their target genes, and their binding motifs were detected within the promoter regions of these functional genes. The top 20 TFs that regulated the largest number of functional genes were associated with 91 functional genes (Figure 6), suggesting that these TFs could contribute to the SI response in *A. trifoliata*.

## 4. Discussion

SI limits pure-line breeding, mating partners, and pollination success in crops, significantly hindering breeding progress and increasing cultivation and management costs [1,14,44]. Therefore, self-compatibility is considered a desirable trait for increasing yield [45], making the elucidation of SI molecular mechanisms and the development of regulatory technologies central goals in the genetic improvement of modern crops. Understanding the molecular basis and regulatory network of SI is fundamental for overcoming SI barriers. For example, the *S-RNase*-regulated GSI mechanism in crops such as potato and apple has been successfully overcome by gene editing and chemical treatment [46,47]. However, in *A. trifoliata*, a recently domesticated fruit crop, the regulatory genes and downstream networks involved in LSI remain unknown, severely limiting the breeding and improvement of this species.

In this study, we conducted a comprehensive comparative transcriptomic analysis of the main SI responses in *A. trifoliata* following self- and cross-pollination. We identified metabolic pathways potentially involved in LSI, including the MAPK signaling pathway, plant hormone signal transduction, and ubiquitin-mediated proteolysis, which are commonly associated with SI [24,27,32]. Additionally, we identified 100 TFs that may play regulatory roles in the SI response; these TFs might coordinate the expression of functional genes involved in pollen recognition and growth, potentially contributing to the regulation of LSI. Furthermore, we observed differences in pollen tube growth between self- and cross-pollinated pistils, along with DEGs involved in pollen recognition and pollen tube development, indicating that the LSI response in *A. trifoliata* is closely linked to pollen tube behavior. Taken together, these results provide a conceptual framework for future studies of LSI, offer valuable insights into the molecular processes underlying LSI, and lay a foundation for guiding experimental validation and functional characterization in *A. trifoliata* and other LSI species.

LSI is widely distributed among angiosperms, yet its regulatory systems and molecular mechanisms remain poorly understood [3,6]. In contrast, the regulatory systems and molecular mechanisms of GSI have been extensively studied in core eudicots. In GSI systems mediated by the *S-RNase*/*SLFs* locus, SI is executed through ubiquitin-mediated protein degradation pathways that ultimately determine the fate of pollen tubes [18,19,20,21,22]. In this study, through observations of pollen tubes following self- and cross-pollination and comparative transcriptomic analyses of pistils in *A. trifoliata*, we identified several phenotypic features and downstream regulatory pathways that resemble those reported in *S-RNase*-regulated GSI systems. Given that *S-RNase* genes have so far been identified only in core eudicot lineages [3], the similarities observed in the LSI response of *A. trifoliata*, a basal eudicot species, are more reasonably interpreted as reflecting shared or convergent downstream regulatory processes of SI.

Firstly, after self-pollination, the pollen tubes of *A. trifoliata* can extend near the ovules; however, compared with cross-pollination, the spread of self-pollinated pollen tubes is restricted. This observation indicates that the LSI response may involve regulatory processes within the pollen tube or closely associated with it. Similarly, in *S-RNase*–mediated GSI systems, self-pollinated pollen can also germinate, but incompatible pollen tubes are inhibited from reaching the ovules before successful fertilization [3,21]. Moreover, comparative transcriptome analysis showed that DEGs between self- and cross-pollinated pistils are enriched in pathways such as plant–pathogen interaction, plant hormone signal transduction, MAPK signaling, and ABC transporters. Notably, these pathways are also enriched in the SI response of *S-RNase*–based GSI species, such as pear and sweet cherry [48,49]. In addition, genes involved in ubiquitin-mediated protein degradation, including components of the SCF^SLF^ complex, were up-regulated in self-pollinated pistils. Given the established role of ubiquitination in regulating pollen tube fate in *S-RNase*–mediated GSI [23,50], this observation suggests that ubiquitin-related processes may also be involved in the LSI response of *A. trifoliata*. Taken together, these results support that the LSI of *A. trifoliata* shares certain downstream regulatory features with *S-RNase*–mediated GSI, while the upstream recognition mechanism remains to be elucidated.

In SSI, self-nonself pollen recognition occurs at the beginning of pollen-stigma contact [51], while in GSI, self-nonself pollen recognition occurs in the pollen tube [52]. However, the timing and spatial characteristics of self–nonself recognition in LSI remain unclear. Observations of pollen tube growth in *A. trifoliata* revealed morphological abnormalities in self-pollinated pollen tubes 144 h after pollination, similar to the twisted pollen tubes observed in *Camellia weiningensis*, which also exhibits LSI. This suggests that self–nonself recognition in *A. trifoliata* may occur prior to this stage, with the observed pollen tube morphological abnormalities potentially being the result of this early recognition. The gene expression differences observed early after self- and cross-pollination further support this interpretation. Compared to cross-pollination, 48 h after self-pollination, pistils of *A. trifoliata* showed up-regulated expression of genes involved in pollen recognition, immune defense responses, and ROS production, particularly within the MAPK signaling pathway. Additionally, comparative transcriptome analysis revealed DEGs associated with hormone signaling and ubiquitin-mediated protein degradation within 96 h after pollination. These findings indicate that the pistil begins to respond differently to self and nonself pollen at relatively early stages after pollination.

Additionally, several studies have shown that SI requires distinguishing self from nonself pollen, a process conceptually similar to self–nonself recognition in plant innate immunity and pathogen defense [45,53]. For instance, the *SRK*, which regulates SSI in *Brassicaceae*, is a member of the receptor-like kinase (RLK) family involved in immune defense [54]. Similarly, genes from the T2 RNase family, to which *S-RNases* belong, have been implicated in defense responses [55]. Early DEGs associated with plant immunity have been reported in *S-RNase*-mediated SI in species such as pear [48]. In *A. trifoliata*, we also observed a similar phenomenon, where DEGs from both self- and cross-pollinated pistils were highly enriched in plant-pathogen interaction pathways at multiple time points after pollination. Although the molecular network linking LSI and immune responses remains unclear, these findings suggest a potential connection between LSI and plant immune defense responses. Future investigations into the mechanisms of LSI could further explore the role of immune defense pathways.

## Figures and Tables

**Table 1:** Expression of ubiquitination complex components.

Component of the Ubiquitination Complex	Gene ID	48 h ^1^	96 h ^2^	144 h ^3^	192 h ^4^	240 h ^5^
SCF complex—Cullin	*EVM0017326*	1.180	\	\	\	\
SCF complex—F-box	*EVM0008054*	2.955	\	\	\	\
SCF complex—F-box	*EVM0008140*	3.076	\	\	\	\
SCF complex—F-box	*EVM0011380*	1.350	\	\	\	−2.901
SCF complex—F-box	*EVM0017111*	2.294	\	\	\	−6.071
SCF complex—F-box	*EVM0020737*	1.763	\	\	\	−1.923
SCF complex—F-box	*EVM0010756*	\	1.400	\	\	\
SCF complex—F-box	*EVM0001176*	\	2.417	−3.233	\	−4.065
SCF complex—F-box	*EVM0006932*	\	1.709	−1.513	\	\
SCF complex—F-box	*EVM0009964*	\	−1.170	\	\	\
SCF complex—F-box	*EVM0019563*	\	2.146	\	\	−3.807
SCF complex—F-box	*EVM0023627*	\	−1.221	\	\	\
SCF complex—F-box	*EVM0000308*	\	\	\	\	−3.864
SCF complex—F-box	*EVM0007772*	\	\	\	\	−10.616
SCF complex—F-box	*EVM0022764*	\	\	\	\	−1.841
E3 (Ubiquitin ligase)—CHIP	*EVM0020660*	1.789	\	\	\	−2.869

^1, 2, 3, 4, 5^ Log (fold change) values of DEGs in the self-pollination pistils compared with the cross-pollination pistils at 48, 96, 144, 192, and 240 h.

**Table 2:** DEGs related to pollen recognition and pollen tube growth and development following self-pollination and cross-pollination.

Gene ID	GO Annotation	48 h ^1^	96 h ^2^	144 h ^3^	192 h ^4^	240 h ^5^
*EVM0004822*	recognition of pollen	2.270	/	−1.583	/	/
*EVM0007051*	recognition of pollen	2.173	−1.966	/	/	−2.445
*EVM0007875*	recognition of pollen	1.034	/	/	/	/
*EVM0008949*	recognition of pollen	1.093	/	/	/	/
*EVM0011721*	recognition of pollen	1.021	/	/	/	/
*EVM0011788*	recognition of pollen	1.360	/	/	/	/
*EVM0013989*	recognition of pollen	1.876	−1.813	/	/	/
*EVM0017407*	recognition of pollen	2.422	/	−1.125	/	−2.041
*EVM0020831*	recognition of pollen	2.213	/	/	/	/
*EVM0021828*	recognition of pollen	1.494	/	/	/	/
*NewGene_4634*	recognition of pollen	−1.978	/	/	/	1.216
*NewGene_8113*	recognition of pollen	1.500	/	/	/	/
*EVM0001209*	recognition of pollen	/	−1.807	/	/	1.001
*EVM0007595*	recognition of pollen	/	2.157	/	/	/
*EVM0011704*	recognition of pollen	/	−2.083	/	/	/
*EVM0012428*	recognition of pollen	/	1.734	−1.064	/	/
*EVM0017272*	recognition of pollen	/	2.063	/	−1.927	−1.813
*EVM0018198*	recognition of pollen	/	−1.511	/	/	/
*EVM0019686*	recognition of pollen	/	2.123	/	/	/
*EVM0021803*	recognition of pollen	/	−1.319	/	/	/
*EVM0023864*	recognition of pollen	/	−1.017	/	/	/
*NewGene_4629*	recognition of pollen	/	1.280	/	/	/
*NewGene_7649*	recognition of pollen	/	−1.520	/	/	/
*EVM0001319*	recognition of pollen	/	/	/	/	−1.243
*EVM0000725*	pollen tube growth/development	1.493	/	/	/	/
*EVM0006406*	pollen tube growth/development	1.104	/	/	/	/
*EVM0020745*	pollen tube growth/development	−1.047	/	/	/	/
*EVM0012853*	pollen tube growth/development	/	−1.014	/	/	/
*EVM0022895*	pollen tube growth/development	/	−1.247	/	/	/
*EVM0002966*	pollen tube guidance	1.021	2.044	−1.192	/	−1.080
*EVM0011431*	regulation of pollen tube growth	/	1.480	/	/	/

^1, 2, 3, 4, 5^ Log (fold change) values of DEGs in the self-pollination pistils compared with the cross-pollination pistils at 48, 96, 144, 192, and 240 h.

## Data Availability

The data presented in this study are available in the National Genomics Data Center at https://www.cncb.ac.cn/ under the project accession number PRJCA019245, with run numbers CRR857369–CRR857398 representing 30 sample datasets.

## Supplementary Materials

The following supporting information can be downloaded at: https://www.mdpi.com/article/10.3390/cimb48030245/s1.

## Abbreviations

The following abbreviations are used in this manuscript:

LSI	Late-acting self-incompatibility
GSI	Gametophytic self-incompatibility
SSI	Sporophytic self-incompatibility
DEGs	Differentially expressed genes
ROS	Reactive oxygen species
PCD	Programmed cell death
MAPK	Mitogen-activated protein kinase
TFs	Transcription factors
